# Multiple thrombosis during eltrombopag therapy

**DOI:** 10.1002/jha2.185

**Published:** 2021-03-13

**Authors:** Yutaka Shimazu, Junya Kanda

**Affiliations:** ^1^ Department of Hematology Kyoto University Hospital Kyoto Japan; ^2^ Department of Hematology Kyoto University Graduate School of Medicine Faculty of Medicine Kyoto Japan

A 57‐year‐old man was admitted to our hospital with vomiting. He had undergone liver transplantation for nonalcoholic steatohepatitis 2 years earlier. Four months after transplantation, he suffered from immune thrombocytopenia and was treated with prednisolone and eltrombopag, which led to a recovery of his platelet counts toward normal (from 23 to 121 × 10^9^/L). On examination, his abdomen was flat with mild tenderness. Hypoxia was not noted. Blood examination revealed elevated D‐dimer (36.2 μg/ml; normal range <1.0). An enhanced CT of the trunk showed multiple thromboembolisms with large infarctions in the liver, spleen and lung (Figure 1 panels A and B, arrowheads indicate thrombosis). Multiple arterial and venous thrombosis in multiple organs were diagnosed. Thrombotic factors, such as antiphospholipid antibody or protein C/S deficiency, were not detected. COVID‐19 was negative. Since both arterial and venous thrombotic events have been reported as adverse events of eltrombopag in 1–2% of cases, we discontinued eltrombopag and initiated heparin treatment. On day 11, dyspnea and severe hypoxia occurred with marked elevation of D‐dimer (138.1 μg/ml). Mechanical ventilation was necessary for respiratory failure due to pulmonary embolism. Anticoagulation therapy was switched from heparin to fondaparinux and he recovered from hypoxia. Following his recovery, oral dual therapy with apixaban and clopidogrel was effective without recurrent thrombosis. Four months after the thrombotic event, his platelet counts remained within the reference range (144 × 10^9^/L) with no additional therapy except for rituximab plus chemotherapy against posttransplant lymphoproliferative disorder, which had been diagnosed after the event.

**FIGURE 1:**
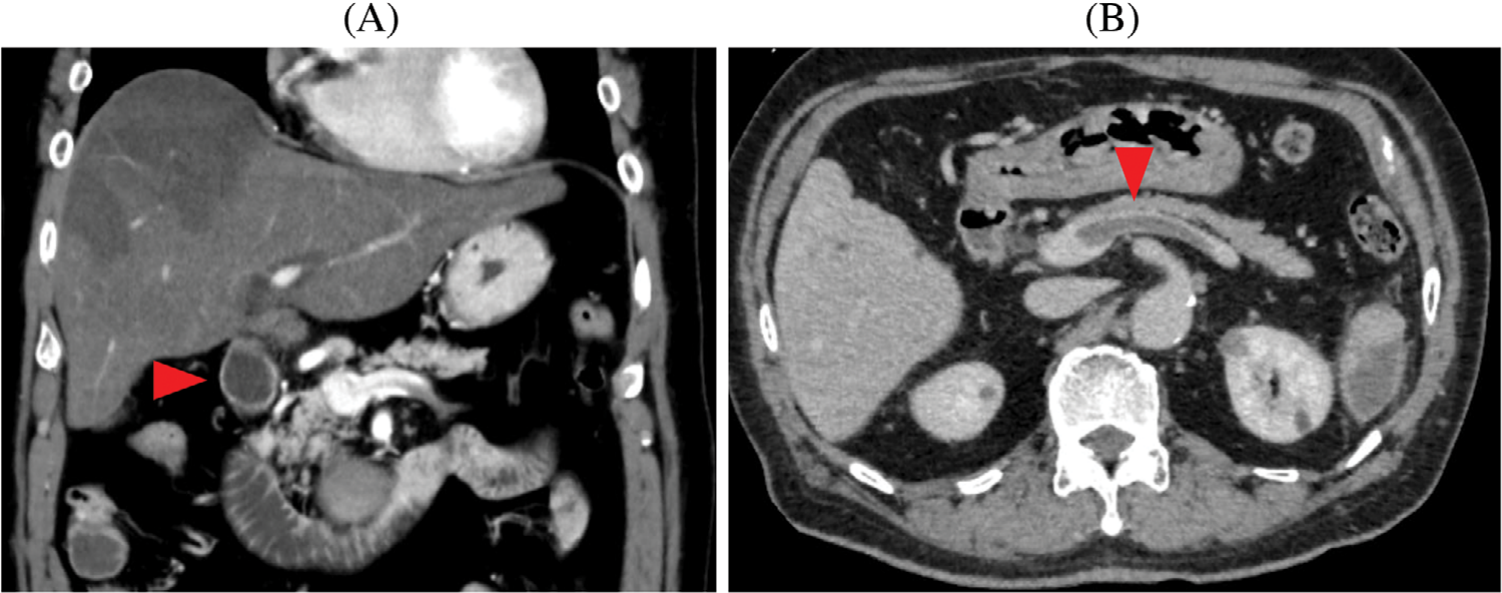
Coronal plane (A) and transverse plane (B) of an enhanced CT of the trunk. Arrowheads indicate thrombosis in the inferior vena cava (A) and the splenic artery (B)

## FUNDING INFORMATION

Takeda Science Foundation.

## CONFLICT OF INTEREST

Yutaka Shimazu declares that there is no conflict of interest.

## ETHICS STATEMENT

All procedures performed in this study involving the patient were in accordance with the ethical standards of our institutional and national research committee, and with the 1964 Helsinki Declaration and its later amendments or comparable ethical standards. Informed consent was obtained from the patient.

